# Homœpathic Life Insurance

**Published:** 1867-09

**Authors:** S. R. Millard

**Affiliations:** Aurora, Ill.


ARTICLE XL.

### HOMŒOPATHIC LIFE INSURANCE.

By S. R. MILLARD, M.D., of Aurora, Ill.

Among the many humbugs that have arisen, in cloudy and mysterious shapes, in the domain of medicine, none has been more vapory, and none is destined to be more evanescent than that known as homœopathy.

It arose in Germany, passed westward, and loomed up in great proportions on the American continent, becoming more and more attenuated as it spread and swelled, till it now no longer casts even a shadow where the light of true medical science falls.

It has always been apparent to us, that those leaving the regular school to practice upon the whims of Hahnemann, were shallow minded and impracticable men, more fitted to be inmates of a lunatic asylum, or a charity hospital, than for the important work of curing the sick, and saving human life. But the extreme of absurd pretension, the very sublimation of folly, was not reached till a few months ago, when a parcel of men in Cleveland organized a Life Insurance Company, for the purpose of *insuring the patrons of homœopathy at rates ten per cent. less than the patrons of scientific medicine.*

For a time, when we read of this lilliputian scheme, we considered it only a joke upon our "sugar pill" neighbors; but before long there came agents and circulars, and ratio and tables, with veritable officers and boards of reference, looking, for all the world, like a real and live life insurance company. Witnessing such a bold and determined movement, we began to question if it could be possible that the lay members of that fantastic school, possessed of more brains and practical sense than the physicians could well and truly run a life insurance company. Before we were done considering this question, and the probable effects of the enterprise upon our own profession and practice; before we could draw our bow and let slip an arrow at the head of the approaching monster, another parcel of men, at Albany, N. Y., set crazy by the brilliant prospects of the Ohio institution, seeing great glory and abundant gains in such an undertaking, got up another company, offering to insure homœopaths for ten per cent. less premium than the patrons of rational medicine. This new concern seemed not quite so ready to cut loose from the old anchorage, but wisely kept half its pigeon holes for old school risks, or, in other words, prepared itself for carrying water conveniently on both shoulders.

Feeling some curiosity as to the interior of life insurance, and the modus operandi of the business, we got manuals, and posted up on the subject, far enough to form some opinion as to the tendency of these new movements. For the first time we learned the nature of mortality tables, of premium reserves, and various kinds of policies. As the result of our investigation we found no cause for alarm. The first company organized, had it received the united and full support of the "sugar pill" people in this country, would possibly have grown to be a power for evil; its influence for a time, at least, would have hurt us. But this second company coming into the field, with its guns more set against the first one than against the old fashioned companies will save us all labor and all care on the subject.

If the Albany company makes up its dividends honestly, in its two sections, we confidently predict that the patrons of regular medicine will receive greater profits, by at least fifteen per cent., than the patrons of homœopathy. Instead, therefore, of being hurt by this new opposition, we expect to be greatly benefited by it. Haman may swing again upon his own gallows.

This whole business but demonstrates anew the utter incompetency of these attenuated minds, to organize or manage any institution of importance. In the absence of a proper education, ignorance and conceit will always lead them into most ridiculous blunders. If they start off apparently well, they soon get at variance, fight each other, and thus destroy what little they might otherwise accomplish.

We now wonder what next? Nothing from the shades of of shadowy moonshine, the mystic realms of homœopathy, can hereafter frighten or even astonish us.

